# Comparison of Waning Antibody Responses After Natural Monkeypox Virus Infection and Mpox Vaccination Beyond 6 Months in South Korea

**DOI:** 10.1093/ofid/ofae566

**Published:** 2024-10-05

**Authors:** So Yun Lim, Hyang Su Kim, Hong Soon Yim, Hyesu Kim, In Ae Han, Doran Yoon, Min-Kyung Kim, Yeonjae Kim, Jihye Um, Gayeon Kim, BumSik Chin, Jun-Sun Park, Jihwan Bang

**Affiliations:** Division of Infectious Diseases, Department of Internal Medicine, National Medical Center, Seoul, Republic of Korea; Public Health Research Institute, National Medical Center, Seoul, Republic of Korea; Public Health Research Institute, National Medical Center, Seoul, Republic of Korea; Public Health Research Institute, National Medical Center, Seoul, Republic of Korea; Division of Infectious Diseases, Department of Internal Medicine, National Medical Center, Seoul, Republic of Korea; Armed Forces Capital Hospital, Seongnam, Republic of Korea; Division of Infectious Diseases, Department of Internal Medicine, National Medical Center, Seoul, Republic of Korea; Division of Infectious Diseases, Department of Internal Medicine, National Medical Center, Seoul, Republic of Korea; Public Health Research Institute, National Medical Center, Seoul, Republic of Korea; Division of Infectious Diseases, Department of Internal Medicine, National Medical Center, Seoul, Republic of Korea; Division of Infectious Diseases, Department of Internal Medicine, National Medical Center, Seoul, Republic of Korea; Public Health Research Institute, National Medical Center, Seoul, Republic of Korea; Division of Infectious Diseases, Department of Internal Medicine, National Medical Center, Seoul, Republic of Korea

**Keywords:** Jynneos, Modified Vaccinia Ankara, monkeypox virus, mpox

## Abstract

Natural Monkeypox virus infection induced significantly higher neutralizing antibody titers than Jynneos vaccination, with similar antibody decay rates beyond 6 months. Jynneos recipients with prior smallpox vaccination showed antibody levels comparable to mpox convalescents.

Mpox, caused by the monkeypox virus (MPXV), is a reemerging zoonotic disease that gained significant attention due to its global outbreak in 2022 [1]. In the early stages of the 2022 outbreak in South Korea, vaccination with Jynneos (Modified Vaccinia Ankara-Bavarian Nordic) was recommended only for individuals with occupational risk of exposure to mpox, administered via subcutaneous injection. However, in early April 2023, South Korea experienced a significant increase in indigenous mpox cases without overseas travel history. This led to a nationwide vaccination campaign in May 2023, targeting high-risk populations with 0.1-mL intradermal administration of Jynneos [2].

Epidemiological data during the 2022 outbreak showed varying effectiveness of the Jynneos against mpox, ranging from 66% to 90% after 2-dose vaccination [3, 4]. In addition, reports of mpox reinfection or breakthrough infection following complete vaccination suggest waning immunity over time [5]. However, there are limited data comparing the immune responses against MPXV after the natural infection and vaccination in terms of long-term immunity [6–8]. Thus, we aimed to compare the antibodies titer beyond 6 months between individuals who recovered from natural MPXV infection and infection-naive individuals who received the 2-dose Jynneos vaccination.

## METHODS

### Study Design and Participants

This prospective study was conducted at the National Medical Center, in Seoul, South Korea, where most patients with mpox were hospitalized. We prospectively enrolled patients with mpox infection who were admitted to our hospital between September 2022 and September 2023. We also recruited healthcare workers and visitors of outpatient clinics, including those from high-risk groups [9] who received the 2-dose Jynneos vaccine as part of the nationwide mpox vaccination campaign, without a history of mpox infection, from December 2023 to January 2024.

The route of Jynneos vaccination was determined based on the target group in South Korea: healthcare workers received a 0.5-mL subcutaneous administration, whereas the high-risk group received a 0.1-mL intradermal injections as part of a dose-sparing strategy. However, subcutaneous vaccination was permitted in cases where there was a history of keloids or if the individual preferred it. Blood samples were planned to be collected at 3-month intervals up to 1 year after either symptom onset (infection group) or the second dose of the vaccine (vaccination group). The definitions for clinical information collected in our study are further described in the Supplementary Materials.

### Patient Consent Statement

Written informed consent was obtained from all patients involved in this study. This study was reviewed and approved by the institutional review board (IRB) of the National Medical Center (IRB nos. 2202-06-069 and 2023-12-139).

### Measurement of Antibody Responses

Neutralizing antibody levels were determined by means of a plaque-reduction neutralization test using MPXV (clade IIb, C.1 lineage) isolated from a patient infected during the outbreak in South Korea in 2023. The detailed protocol is described in the Supplementary Materials.

### Statistical Analysis

We used χ^2^ or Fisher exact tests to compare categorical variables and Student *t* or Mann-Whitney *U* tests compare continuous variables, as appropriate. We estimated a linear mixed model including a random effect intercept for comparison of antibody responses between the different groups. The time scale used was months from exposure to viral antigen, either by infection (symptom onset) or second-dose vaccination. We included data from 6 to 12 months after the last exposure to the viral antigen for the analysis. An additional analysis within the vaccinated group was performed using data up to 9 months to ensure balanced data across the groups. All tests of significance were 2-tailed, and differences were considered significant at *P* < .05. Data were analyzed using R software, version 4.1.3 (R Project for Statistical Computing).

## RESULTS

The final analysis included a total of 48 vaccine recipients and 14 individuals with previous infections who were followed up for >6 months after vaccination or infection. Ten convalescent participants (71%) and 26 vaccinees (54%) had human immunodeficiency virus (HIV) infection (*P* = .25). No other immunocompromising conditions were identified. All but one participant with HIV were on antiretroviral therapy at the time of the study. The demographic and clinical characteristics of the study participants are presented in Table 1.

**Table 1.** Demographic and Clinical Characteristics of Study Participants

Variable	Participants, No. (%)^a^		*P* Value
	Previous Infection (n = 14)	Vaccination (n = 48)	
Age, median (IQR), y	33 (30–35)	35 (32–46)	.09
Male sex	14 (100)	38 (79)	.10
PLWH	10 (71)	26 (54)	.25
CD4 cell count, medium (IQR), cells/µL	487 (344–523)	610 (448–709)	.09
Undetectable HIV RNA	8 (80)	26 (100)	.07
Time from last exposure to viral antigens, median (IQR), mo^b^	9.4 (8.9–9.9)	6.8 (6.2–8.3)	<.001
Previous smallpox vaccination^c^	1 (7)	13 (27)	.16
Vaccination group			
Intradermal vaccination	NA	29 (60)	NA
Interval between 2 vaccine doses, median (IQR), d	NA	30 (28–36)	NA
Infection group			
Clade IIb, C.1 lineage^d^	9 (64)	NA	NA
Unknown^e^	5 (36)	NA	NA
Severe mpox case	1 (7)	NA	NA
Tecovirimat use	8 (57)	NA	NA
Death	0	NA	NA

Abbreviations: HIV, human immunodeficiency virus; IQR, interquartile range; NA, not applicable; PLWH, people living with HIV.

^a^Data represent no. (%) of participants unless otherwise indicated.

^b^Time since symptom onset (infection group) or second vaccine dose (vaccination group).

^c^Born in 1979 or earlier (ie, during the era of a nationwide smallpox vaccination program in South Korea).

^d^Virus clade/lineage identified through whole-genome sequencing.

^e^Lineage was unidentified for 5 cases; only convalescent samples were available.

**Figure 1.**
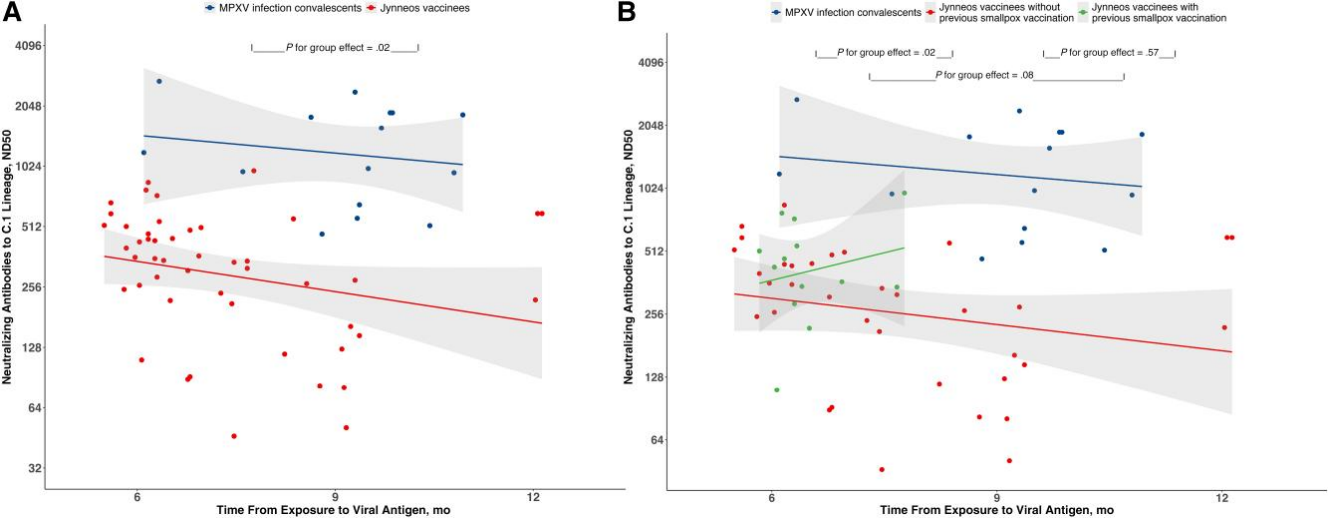
Antibody titers in mpox virus (MPXV) infection convalescents and Jynneos vaccinees (*A*) and in MPXV infection convalescents and Jynneos vaccinees with or without previous smallpox vaccination (*B*). Abbreviation: ND50, 50% neutralization dose.

In a linear mixed-effect model comparing neutralizing antibody titers between convalescents from mpox and Jynneos vaccinees, we found a significant group effect (*P* = .02), with the MPXV infection group having higher neutralizing antibody titers than the vaccination group. However, there was no significant time effect (*P* = .17 for the infection group and *P* = .40 for the vaccination group), suggesting that the antibody titers did not change significantly over time in each group. In addition, the group-by-time interaction was not statistically significant (*P* = .38), indicating that the difference in antibody titers between the 2 groups did not vary significantly over time (Figure 1*A*).

In addition, we compared neutralizing antibody titers among 3 groups: MPXV infection convalescents, Jynneos vaccinees without previous smallpox vaccination, and Jynneos vaccinees with previous smallpox vaccination. Only one individual with a previous MPXV infection had received a smallpox vaccination. Specifically, Jynneos vaccinees without previous smallpox vaccination had significantly lower antibody titers than the MPXV infection convalescents (*P* = .02). Jynneos vaccinees with previous smallpox vaccination also showed antibody titers comparable to those in the MPXV infection convalescents, but this was not statistically significant (*P* = .08) (Figure 1*B*).

Furthermore, we investigated the potential impact of HIV infection on the neutralizing antibody titers among vaccine recipients. We found no differences in antibody levels between participants with or without HIV infection among Jynneos vaccinees (*P* = .36; Supplementary Figure 1 and Supplementary Table 1). In addition, no significant group-by-time interaction was identified between the 2 groups (*P* = .33). Remarkably, intradermal vaccination, the main route for the high-risk group, also did not show a significant difference in neutralizing antibody titers compared with subcutaneous vaccination (*P* = .61; Supplementary Figure 2 and Supplementary Table 2), with no significant group-by-time interaction between the 2 groups (*P* = .48).

## DISCUSSION

Only a few previous studies compared the immune responses against MPXV elicited by infection and vaccination in terms of long-term immunity [6, 7]. We found that natural MPXV infection induced higher neutralizing antibody levels against the MPXV C.1 lineage than those induced by the 2-dose Jynneos vaccination beyond 6 months; vaccinees with a history of smallpox vaccination had antibody levels comparable to those of MPXV infection convalescents.

Our study had 3 important findings. First, Jynneos vaccinees with prior smallpox vaccination had neutralizing antibody levels comparable to MPXV infection convalescents, while those without prior smallpox vaccination had significantly lower levels. These findings are consistent with the previous studies showing high neutralizing antibodies against MPXV in individuals with childhood smallpox vaccination after receiving the Jynneos vaccine [7, 10, 11], likely due to the cross-neutralizing activity of the historic smallpox vaccination.

In addition, Jynneos vaccine recipients with HIV infection showed neutralizing antibody levels comparable to levels in those without HIV infection in our study, despite previous studies showing variable effects of HIV infection on antibody responses after vaccination [6, 12]. This observation may be explained by several factors. The median CD4 T-cell count was 610/µL (interquartile range, 448–709/µL), with undetectable HIV RNA in all participants, suggesting minimal impact of HIV status on antibody response. Remarkably, we observed no significant difference in neutralizing antibody responses among participants with HIV infection when stratified by CD4 cell count, using a threshold of 350/μL (*P* = .45). Another consideration is that asymptomatic or mild MPXV infections may have been underreported in participants with HIV infection, given that a significant number of mpox cases were reported in people living with HIV. Moreover, the impact of prior smallpox vaccination on antibody levels against MPXV was also observed in people living with HIV [11], outweighing the influence of HIV status.

Finally, no significant difference in neutralizing antibody levels was found based on the route of vaccine administration. Both subcutaneous and intradermal administration of Jynneos demonstrated comparable real-world effectiveness in epidemiological data [3] and similar antibody responses in immunological data [12]. Since the route of vaccination was determined based on the target group in South Korea (healthcare workers received subcutaneous injections, whereas the high-risk group received intradermal injections), the neutralizing antibody levels between different routes of vaccination may be explained by similar factors observed in participants with HIV infection. However, recent epidemiological data presented a significantly longer median interval between the second vaccine dose and illness onset for individuals who received intradermal doses than in those with subcutaneous doses, despite the unclear implications [13]. Therefore, further studies are necessary to elucidate the underlying immunological mechanisms and long-term effects of the vaccine administration route.

Our study was limited by the small sample size of the participants with previous MPXV infection, due to the challenging follow-up after discharge, possibly influenced by their sociodemographic backgrounds. Of note, the presence of only one severe case in our study limited the comparison of antibody responses based on disease severity, warranting further investigation. In addition, we did not measure T-cell responses. Finally, there may have been unreported or undetected asymptomatic or mild MPXV infection among vaccine recipients, potentially influencing the result of antibody responses after vaccination.

In conclusion, our data suggest that natural MPXV infection induces more robust antibody responses than 2-dose Jynneos vaccination beyond 6 months. Previous smallpox vaccination might be an associated factor for higher neutralizing antibodies after vaccination.

## Supplementary Material

ofae566_Supplementary_Data

## Supplementary Data

Supplementary materials are available at *Open Forum Infectious Diseases* online. Consisting of data provided by the authors to benefit the reader, the posted materials are not copyedited and are the sole responsibility of the authors, so questions or comments should be addressed to the corresponding author.
