# Supplementary material for: Comparison of Waning Antibody Responses After Natural Monkeypox Virus Infection and Mpox Vaccination Beyond 6 Months in South Korea
Source: Open Forum Infect Dis. 2024 Oct 5;11(10):ofae566. doi: 10.1093/ofid/ofae566 (PMC11500191; doi:10.1093/ofid/ofae566)
Supplement: ofae566_Supplementary_Data [file ofae566_supplementary_data.docx]

**Supplementary Materials**

| **Detailed Methods** ...........……........……..................……........……............................ | p. 2 |
| --- | --- |
| **Supplementary Tables** …...........……........……........……........……........................... | p. 4 |
| **Supplementary Figures** …...........……........……........……........……......................... | p. 6 |

**Detailed Methods**

***Definitions***

A case of Mpox was identified based on a positive polymerase chain reaction (PCR) assay result from any clinical samples (skin, oropharyngeal swab, blood, anogenital lesion, and others). Severe Mpox cases were defined as previously described. [1] Among the study participants in the vaccination group, all received the Jynneos vaccine. According to the Korean government's policy, healthcare workers were administered the vaccine via the subcutaneous route, whereas the high-risk group received the intradermal vaccination, except in special cases such as individuals with a tendency to develop keloids. Smallpox vaccination refers to the childhood vaccination of individuals born in 1979 or earlier, during the era when a nationwide smallpox vaccination program was being implemented in South Korea.

Demographic characteristics, underlying immunocompromising diseases, history of Jynneos (Modified Vaccinia Ankara, Bavarian Nordic) vaccination, treatment of Mpox infection, and outcomes were reviewed. In participants with human immunodeficiency virus type 1 (HIV-1) infection, baseline CD4+ T-cell and HIV-RNA test results were also reviewed. Specifically, for those with Mpox infection, CD4+ T-cell and HIV-RNA tests were conducted at admission, whereas for vaccinees, the most recent results within 6 months prior to the first dose of Jynneos were identified. Undetectable HIV-RNA was defined as <50 copies/mL. Time from exposure to viral antigen was defined as the duration from symptom onset for previously infected individuals or from the second dose administration for vaccinated individuals to sample collection.

***Measurement of antibody responses***

Neutralizing antibody levels were determined by a plaque-reduction neutralization test (PRNT) using MPXV C.1 lineage isolated from a patient infected in a domestic community outbreak in South Korea in 2023. Serial two-fold dilutions of plasma samples, heat-inactivated at 56°C for 30 minutes, were incubated with 80-100 PFU of virus for 1 hour at 37°C and then applied to VeroE6 cells at a concentration of 2×10^5^/mL. After a one-hour incubation, the cells were overlaid with 0.5% agarose in MEM with 5% FBS and antibiotics, and incubated for 5 days at 37°C in a 5% CO_2_. When plaques are observed, the cells were fixed with a 4% formaldehyde solution for 30 minutes and stained with a 1% crystal violet solution. The 50% neutralization dose (ND50) was calculated using the Kärber formula, with ND50 values greater than 20 considered positive. All samples, including positive controls (virus only) and negative controls (media only), were tested in duplicate. All experimental protocols involving live viruses were conducted following the standard operating procedures for Biosafety Level 3 laboratories.

**Supplementary Table 1**. **Clinical characteristics of the vaccine recipients according to the HIV infection**

| **Variable** | **No HIV infection**  **(n=19)** | **HIV infection**  **(n=26)** | ***P* value** |
| --- | --- | --- | --- |
| Age (years) | 34 (28−41) | 38 (33−48) | 0.046 |
| CD4 cell count (cells/mm^3^) | NA | 610 (448−709) |  |
| Previous smallpox vaccination^*^ | 3 (16) | 10 (38) | 0.185 |
| Intradermal vaccination | 3 (16) | 26 (100) | <0.001 |

*Born in 1979 or earlier, i.e., during the era of a nationwide smallpox vaccination program in South Korea

**Supplementary Table 2. Clinical characteristics of the vaccine recipients according to the vaccination route**

| **Variable** | **Intradermal**  **(n=29)** | **Subcutaneous**  **(n=16)** | ***P* value** |
| --- | --- | --- | --- |
| Age (years) | 37 (33−47) | 32 (28−40) | 0.069 |
| HIV infection | 26 (89.7) | 0 | <0.001 |
| Previous smallpox vaccination^*^ | 11 (37.9) | 2 (12.5) | 0.145 |

*Born in 1979 or earlier, i.e., during the era of a nationwide smallpox vaccination program in South Korea


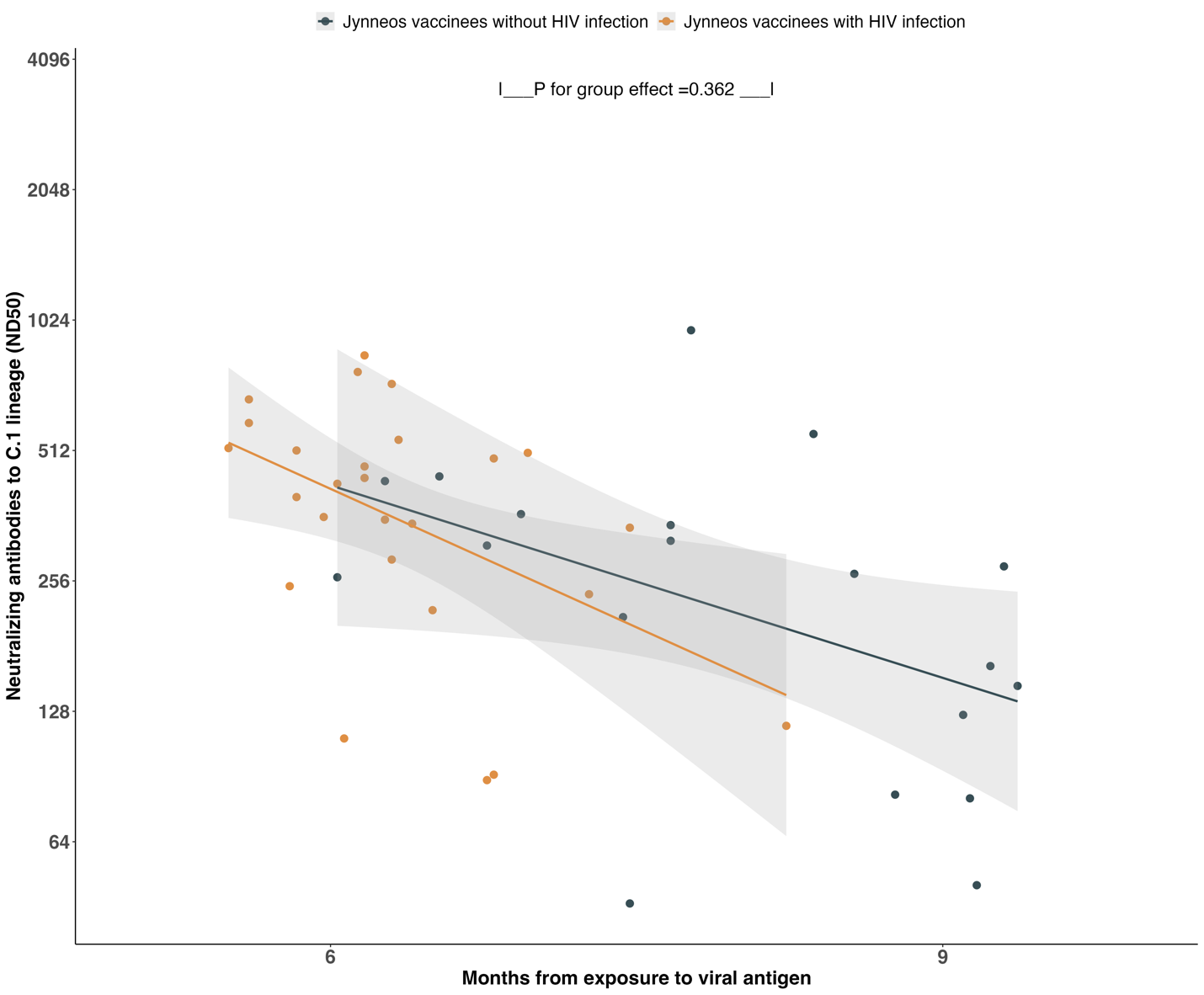


**Supplementary Figure 1. Neutralizing antibodies against Mpox after Jynneos vaccination according to the HIV-1 infection**


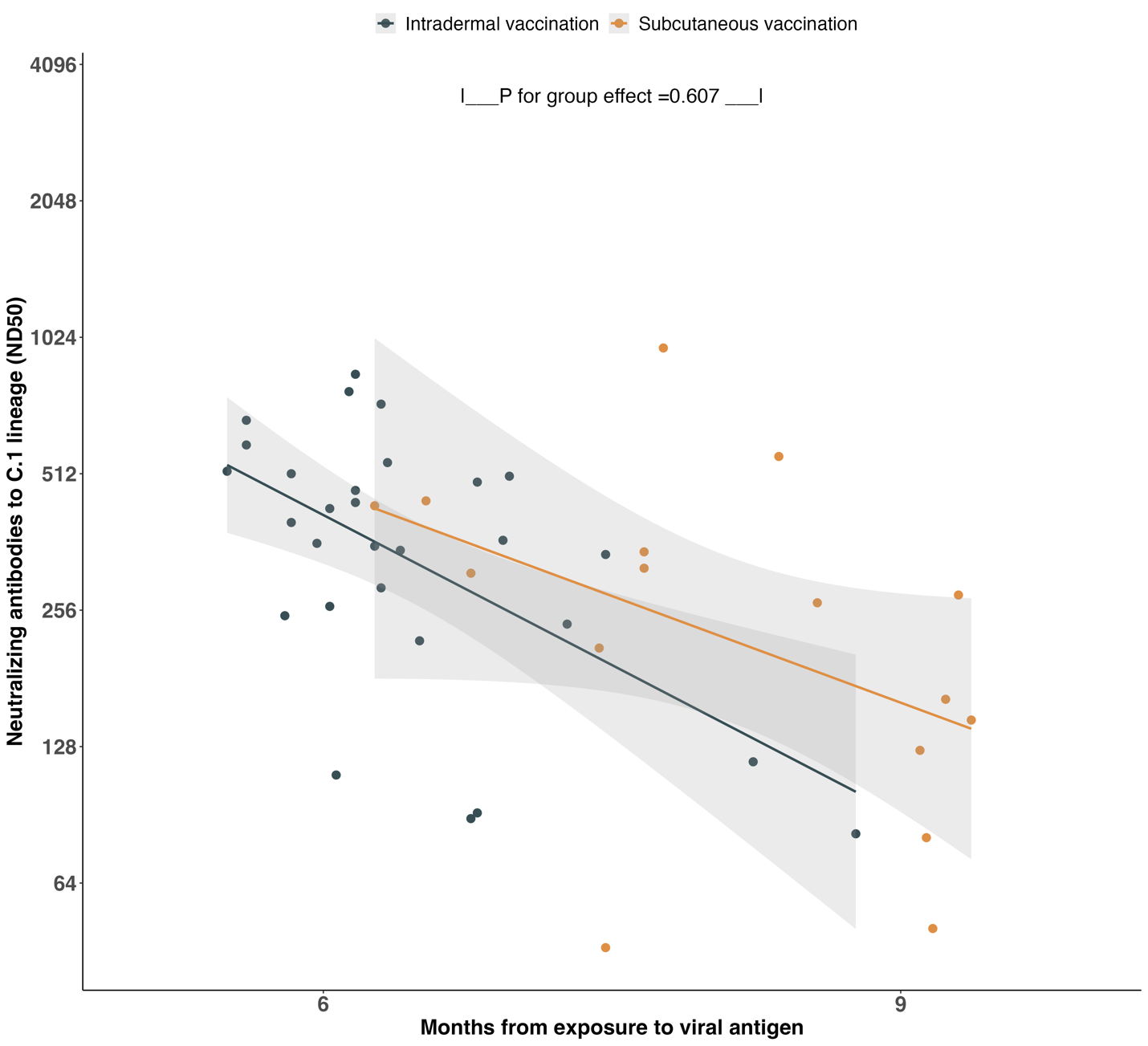


**Supplementary Figure 2.** **Neutralizing antibodies after Jynneos vaccination according to the vaccination route.**

**References for Supplementary Materials**

1. Network CHA. Severe Manifestations of Monkeypox among People who are Immunocompromised Due to HIV or Other Conditions. Available at: https://emergency.cdc.gov/han/2022/han00475.asp. Accessed May 31.
